# Supplementary material for: Scalable Bayesian inference for bradley–Terry models with ties: an application to honour based abuse
Source: J Appl Stat. 2024 Dec 11;52(9):1695–712. doi: 10.1080/02664763.2024.2436608 (PMC12217112; doi:10.1080/02664763.2024.2436608)
Supplement: Mapping Risk of Female Genital Mutilation in South Yorkshire.pdf [file CJAS_A_2436608_SM7027.pdf]

UNIVERSITY OF BIRMINGHAM

Mapping Risk of Female Genital Mutilation in South Yorkshire

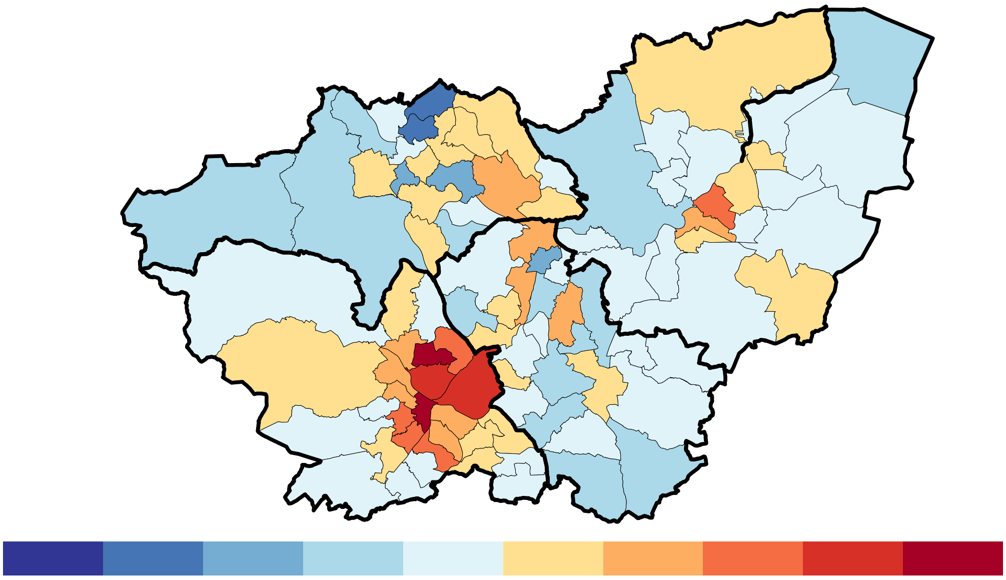

Dr Rowland Seymour, Fabian Hernandez, Prof Caroline Bradbury-Jones, Mike Allaway
January 2023

Using a new comparative judgement method, researchers at the University of Birmingham have mapped data on the risk of Female Genital Mutilation (FGM) to ward level data pertaining to South Yorkshire. The map above shows the risk of FGM in each ward in the county. Red wards have the highest risk and blue wards have the lowest risk.

To estimate the risk of FGM, the research team carried out a comparative judgement study. Comparative judgement is a new method to estimate risk of human rights abuses and has previously been used to estimate deprivation levels at local levels in developing countries and forced marriage in the UK (more information about the research methodology can be found in Seymour et. al. (2022)).

In this study, 18 experts in safeguarding against FGM from across South Yorkshire were shown pairs of wards and asked which of the pair has a higher prevalence of FGM. It is often easier to compare areas than to rank them outright or to place areas of a scale of low/high risk. The participants provided us with 877 comparisons of pairs of wards in the county. From all the comparisons, it was then possible to rank the wards from highest to lowest risk of FGM.

Overview

Female Genital Mutilation (FGM) is a procedure where the female genitals are deliberately cut, injured or changed, but there is no medical reason for this to be done.

By collecting data from front line safeguarding professionals in South Yorkshire, researchers at the University of Birmingham have mapped data on the risks of FGM to different wards in the county.

The research team found that inner city wards in Sheffield have the highest risk of FGM. Town ward in Doncaster is also in the top ten wards with the highest risk. The lowest risk wards are St Helens, Royston and Stairfoot.

Recommendations

Based on the data and analysis, the research team recommend:

1. Agencies working in inner city wards in Sheffield are alerted to the risk of FGM in this area.
2. Agencies working in Doncaster Town are alerted to the risk of FGM in this area.
3. Further research is carried out to understand the drivers of FGM.

Risk of FGM by Local Authority

The plot below shows the risk of FGM in each ward grouped by local authority. When estimating the risk of FGM, the research team set the average risk across the whole county to be zero. Wards with a large negative value have the highest risk of FGM. Wards with a large positive value have the lowest risk of FGM.

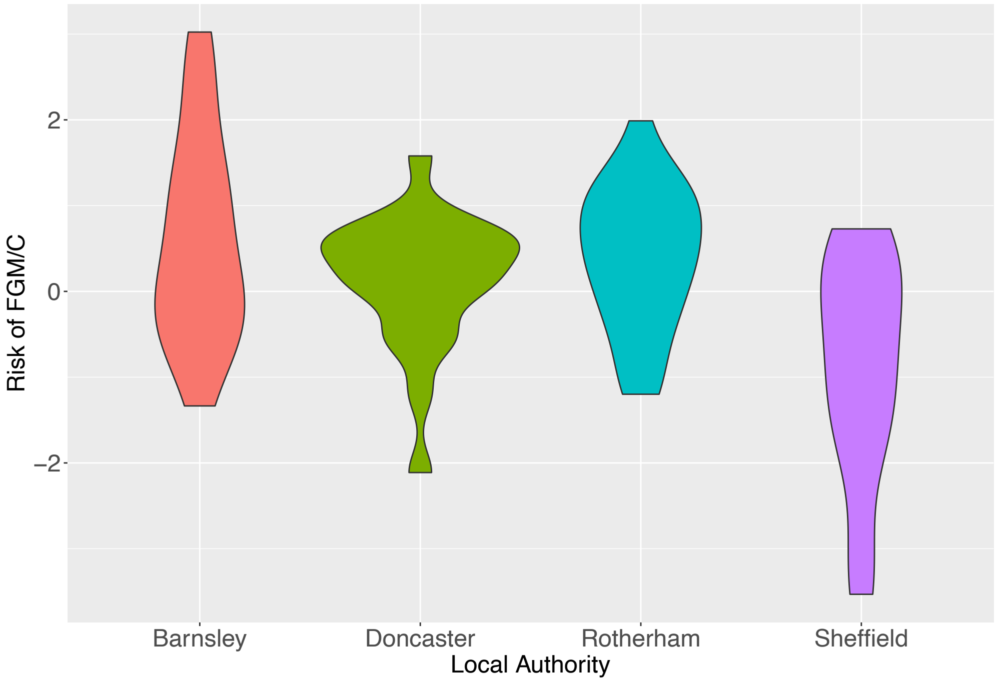

Sheffield City Council has the wards with the highest risk of FGM. Two thirds of the city's wards are estimated to have a risk higher risk than the average ward in South Yorkshire. The majority of wards in Barnsley are estimated to have low risk of FGM, with Darfield and Cudworth being the exception. The majority of wards in Rotherham are estimated to have risk that is about average across South Yorkshire. The risk of FGM in Doncaster is concentrated in Doncaster Town, with the rest of the wards in the local authority having average risk for South Yorkshire.

The map on the right shows the uncertainty in the estimates for the risk in each ward. Wards shown in dark red have the highest uncertainty in their estimates, and pale-yellow wards have the lowest. Across the county, the estimates have low uncertainty, although there are patches in Barnsley, where the team were only able to collect a small number of comparisons. This explains why the uncertainty is larger for the risk of FGM in Doncaster.

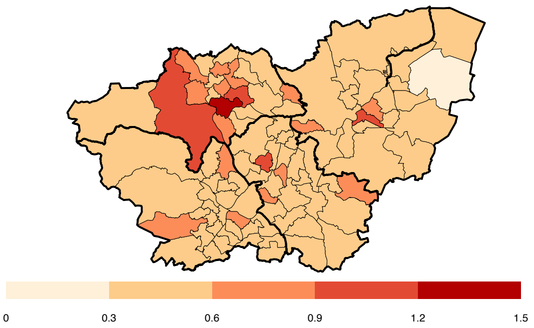

10 Wards with the highest risk of FGM

We estimate the ten wards with the highest risk of FGM are:

1. City (Sheffield)
2. Firth Park
3. Darnall
4. Burngreave
5. Town (Doncaster)
6. Shiregreen and Brightside
7. Nether Edge and Sharrow
8. Gleadless Valley
9. Broomhill and Sharrow Vale
10. Hillsborough

Why is this important?

At present, there are no ward or county level estimates of the prevalence and risk of FGM. This limits the development of local policies to tackle FGM and protect potential victims. Mapping FGM risk enables practitioners to advocate for increased funding, services, and training for professionals in the most at-risk areas to prevent and safeguard victims of FGM.

Acknowledgements

This work was supported by the University of Birmingham School of Mathematics. Digital Research Support was provided by the Birmingham Environment for Academic Research. South Yorkshire Police support with data collection.

UNIVERSITY OF BIRMINGHAM
Edgbaston, Birmingham, B15 2TT, United Kingdom
www.birmingham.ac.uk
