# Supplementary material for: Scalable Bayesian inference for bradley–Terry models with ties: an application to honour based abuse
Source: J Appl Stat. 2024 Dec 11;52(9):1695–712. doi: 10.1080/02664763.2024.2436608 (PMC12217112; doi:10.1080/02664763.2024.2436608)
Supplement: Supplementary Material.pdf [file CJAS_A_2436608_SM7026.pdf]

Supplementary Material

1 Simulation Study

To test our MCMC algorithm's ability to efficiently scale, we ran a simulation study, as described in the Section 4.1 of the main text. For the Metropolis-Hastings Random Walk representation, the average effective sample size per second for the risk parameters $\boldsymbol{\lambda}$ across all simulations was 0.035 (0.070 in the most efficient simulation and 0.006 in the least). The Pólya-Gamma latent variable representation offers a marked improvement, with an average effective sample size per second for the risk parameters of 108 (121 in the most effective simulation, and 91.1 in the least). As both representations use a Metropolis-Hastings Random Walk algorithm to sample values for the tie parameter δ, we saw a smaller but still considerable difference. The average effective sample size per second across all simulations for δ was 13.6 (34.4 in the most effective simulation, and 2.60 in the least) using the Metropolis-Hastings Random Walk representation and 19.1 (29.2 in the most effective simulation, and 5.77 in the least).

We fitted the model using the Pólya-Gamma latent variable representation and the Metropolis-Hasting Random Walk algorithm proposed in [1]. For the Pólya-Gamma latent variable representation, we run the MCMC algorithm for 5,000 iterations removing the fist 100 iterations as a burn-in period. For the Metropolis-Hastings Random Walk algorithm, we ran the MCMC for 500,000 iterations removing the first 100,000. iterations as a burn-in period.

2 Sensitivity Analyses

2.1 Sensitivity analysis for the variance hyperparameter

When fitting the model to the South Yorkshire data, the 95% credible interval for the variance hyperparameter α^2 was (24.0, 84.5), with a posterior median of 44.2. A trace plot and histogram of the posterior distribution are shown in Figure 1. To assess how sensitive the results are to this parameter that varies significantly, we carried out a sensitivity analysis. We fitted the model to the data from South Yorkshire fixing the value of this parameter to represent different spatial correlation scenarios. We assumed *strong* prior correlation ($\alpha^2 = 24$), and *weak* prior correlation ($\alpha^2 = 84$). These values were chosen to match the end points of the 95% credible interval. We also fitted the model fixing this hyperparameter to a value representing *very strong* prior correlation ($\alpha^2 = 1$). We recorded the posterior median and standard deviations of the quality

α^2	2.5%	50%	97.5%
1	0.302	0.355	0.416
24	0.375	0.448	0.526
84	0.410	0.491	0.570
Learned	0.390	0.468	0.552

Table 1: The posterior medians and 95% credible intervals for the tie parameter δ in the sensitivity analysis for the prior variance parameter α^2.

values for each ward and these are shown in Figure 2. We also recorded the posterior median and 95% credible interval for the tie parameter δ and these are displayed in Table 1.

When fitting the model using both the strong and the weak strength parameters, the posterior median values for the quality of each ward are highly similar to when α^2 is learned (Pearson correlation coefficients 0.995 and 0.996 respectively). Comparing the posterior median values from the strong and weak directly, show that these are largely the same, with some slight shrinkage when using the strong prior correlation. When fix the value of α^2 to a value representing very strong prior spatial correlation, we see substantial shrinkage in the results, suggesting the model is oversmoothing the results and estimating that the risk of FGM is similar in each ward. Table 1 shows the posterior distribution is largely unaffected by the value of α^2 except when using very strong prior correlation.

We do see considerable differences in the standard deviations of the posterior distributions for the quality parameters when changing the value of the variance hyperparameter. When using weak prior correlation, the standard deviations are the largest, around 4 units, and this decreases when using the strong and then the very strong prior correlation. The standard deviations of the posterior distributions are a similar size when using the very strong prior correlation and when learning the value of the variance hyperparameter. However, when using the very strong prior correlation, the values of the standard deviations are similar for all wards, suggesting that this value is oversmoothing the results.

Given the results for the posterior median values for the quality and tie parameters when α^2 is fixed the end points of the 95% credible interval and highly similar, it is unlikely that our results are being strongly influenced by the value of α^2 and we are unlikely to be oversmoothing the results. However, we do find that learning the value of α^2 does all for us to reduce the uncertainty in the final estimates for the quality parameter.

2.2 Sensitivity analysis for the tie parameter

To assess how sensitive our model is to the value of the tie parameter δ, we carried out a sensitivity analysis. We simulated sets of comparisons with δ fixed to 0.1, 0.5, 1, and 2. These values were chosen as they result in sets of comparisons where approximately

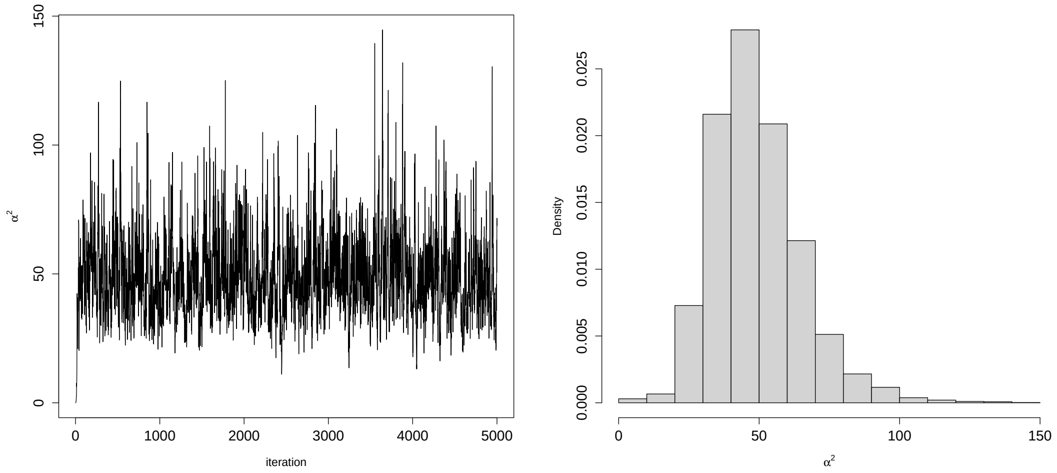

Figure 1: Left: The trace plot for the variance hyperparameter α^2 when fitted to the South Yorkshire data. Right: A histogram of the posterior distribution for α^2.

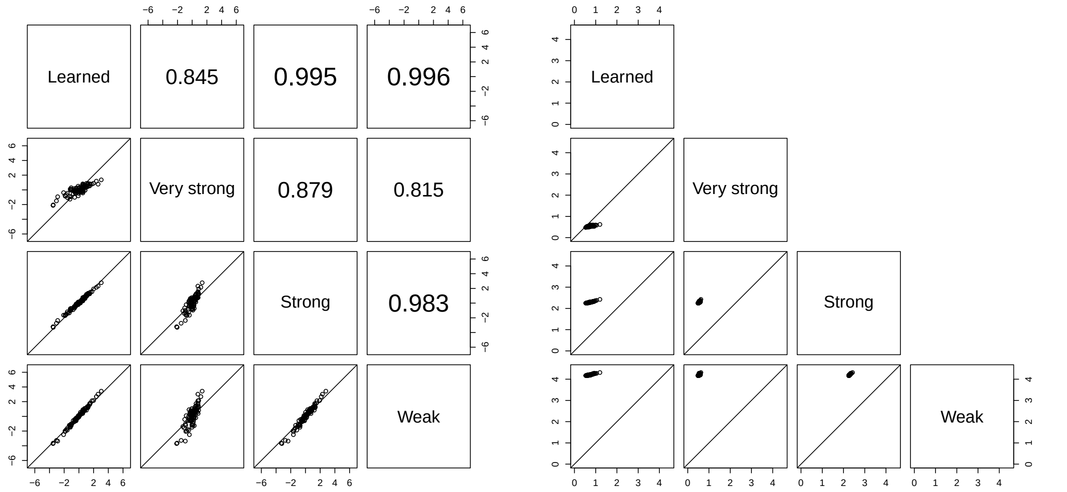

Figure 2: Sensitivity analysis on α^2 showing the values of the quality parameters (left) and standard deviations (right) estimated using the different values for α^2. The diagonal lines in the Figure show the line $y = x$. The Pearson correlation coefficients are shown in the upper part of the Figure for the posterior medians.

δ	Mean absolute error in $\boldsymbol{\lambda}$	Mean relative error in δ
0.1	0.327	0.198
0.5	0.306	0.080
1	0.307	0.059
2	0.342	0.251

Table 2: The mean absolute and relative errors across each 50 synthetic data sets for each value of the tie parameter for δ used in the sensitivity analysis.

5%, 20%, 50% and 75% of the comparisons are tied. For each set of comparisons, we generated a synthetic set of quality parameters for South Yorkshire using the prior distribution with covariance matrix in Equation (12) of the main text. We then generated a set of 800 comparisons using one of the values of δ listed above. For each value of δ, we repeated this process 50 times. We then fit the model to each of the sets of comparisons and infer the model parameters, including the value of the tie parameter and the ward quality parameters.

Table 2 shows the mean absolute error in the both the tie parameter δ and the ward quality parameter $\boldsymbol{\lambda}$. We can see the error for the ward quality parameters is similar for all values of δ, although the error is slightly higher when δ is set to the lowest and highest value. The error in the estimate for δ is again highest when δ is set to the lowest and highest value. This is due to the amount of information the model has to learn about these parameters. When $\delta = 0.1$ around 5% of the comparisons are tied, providing less information for the model to learn about this parameter compared to larger values of δ. The increase in the error for δ is also shown in the ward quality parameters, which the model adjusts to account for the error in the tie parameter. When $\delta = 2$ the opposite situation occurs. As the vast majority of comparisons are ties, the model has less information to learn about the value of the quality parameters and so the error increases. The leads to an increase in the error for the tie parameter. Despite this, the error in the ward quality parameters is similar for all values of δ.

Given that in our real data between 15% - 25% of the comparisons are tied, we believe we can infer the model parameters accurately.

3 Diagnostics for the studies on real data

We carried out model diagnostics to ensure that our MCMC algorithm was performing well. This included viewing trace plots and carrying out convergence diagnoses. We carried out two convergence diagnoses, the first is the Gelman-Rubin statistic, based on fitting the model to the data using five different random seeds. The statistic R, involves computing the variance between the five chains and the variance within each chain, and when the statistic takes values close to 1, it suggests there are no convergence issues. The second is the Geweke's diagnostic, which compares the mean of the chain during the first 100 iterations (the burn-in period) with the last 50% of interactions in the chain. If there is no significant difference between these values, it suggests that the chain has

converged in the first 50 iterations.

3.1 South Yorkshire

We fit the model to the South Yorkshire data set and produce the results shown in Section 4.1 of the main text. Trace plots for λ_{20}, λ_{40}, λ_{60}, and λ_{80} shown in Figure 3. These show that the ward quality parameters converge quickly and mix well. Based on the diagnostic plots, we consider the first 50 iterations as a burn-in period. Figure 4 shows autocorrelation plots for the ward quality parameters, the prior variance parameter and the tie parameter. For the ward quality parameter, the autocorrelation decays almost immediately, and for the prior variance and tie parameters the lag is around 15 and 10 iterations.

Table 3 displays the Gelman-Rubin statistics and the p-value for the Geweke diagnostics for the ward quality parameters λ_{20}, λ_{40}, λ_{60}, λ_{80}, the tie parameter δ and the variance hyperparameter α^2. Both diagnostic checks suggest that the chains for these parameters are converging to the fist 100 iterations can be treated as a burn-in period.

Parameter	R	Geweke p-value
λ_{20}	1.01	0.957
λ_{40}	1.01	0.394
λ_{60}	1.00	0.380
λ_{80}	1.00	0.942
δ	1.15	0.089
α^2	1.04	0.192

Table 3: The values of the MCMC convergence diagnoses for parameters in the model when fitted to the South Yorkshire data set.

Parameter	R	Geweke p-value
λ_{10}	1.02	0.305
λ_{20}	1.02	0.09
λ_{30}	1.02	0.958
λ_{40}	1.00	0.485
δ	1.16	0.237
α^2	1.02	0.078

Table 4: The values of the MCMC convergence diagnoses for parameters in the model when fitted to the Oxfordshire data set.

3.2 Oxfordshire

We fit the model to the Oxfordshire data set and produce the results shown in Section 4.2 of the main text. Trace plots for λ_{10}, λ_{20}, λ_{30}, and λ_{40} shown in Figure 5 (the trace plot for α^2 is shown above and for δ is shown in the main text). These show that the ward quality parameters converge quickly and mix well. Figure 6 shows autocorrelation plots for the ward quality parameters, the prior variance parameter and the tie parameter. For the ward quality parameter, the autocorrelation decays almost immediately, and for the prior variance and tie parameters the lag is around 17 and 15 iterations.

Based on the diagnostic plots, we consider the first 50 iterations as a burn-in period. Table 3 displays the Gelman-Rubin statistics and the p-value for the Geweke diagnostics for the ward quality parameters λ_{20}, λ_{40}, λ_{60}, λ_{80}, the tie parameter δ and the variance hyperparameter α^2. Both diagnostic checks suggest that the chains for these parameters are converging to the fist 100 iterations can be treated as a burn-in period.

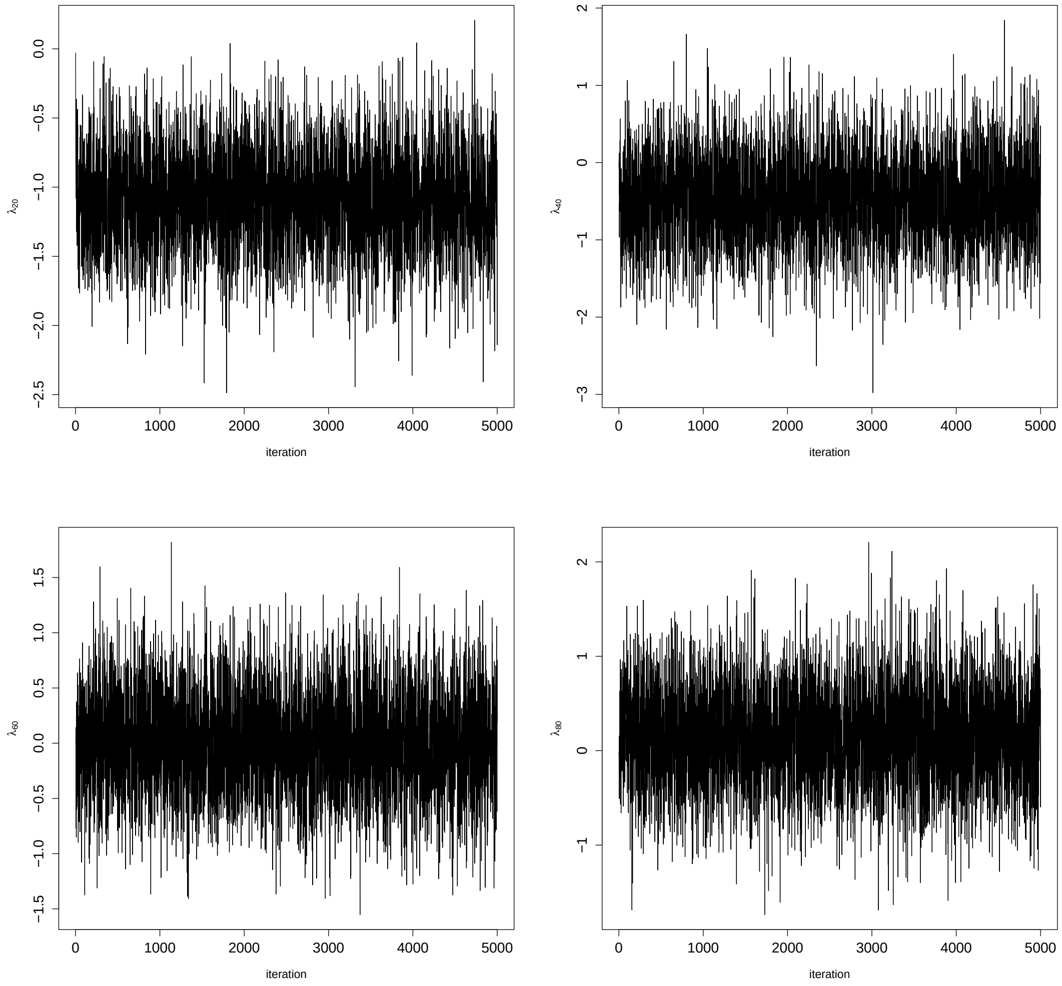

Figure 3: Trace plots for the quality parameters for wards 20, 40, 60, and 80 in the South Yorkshire study. All iterations are shown, including the burn-in period.

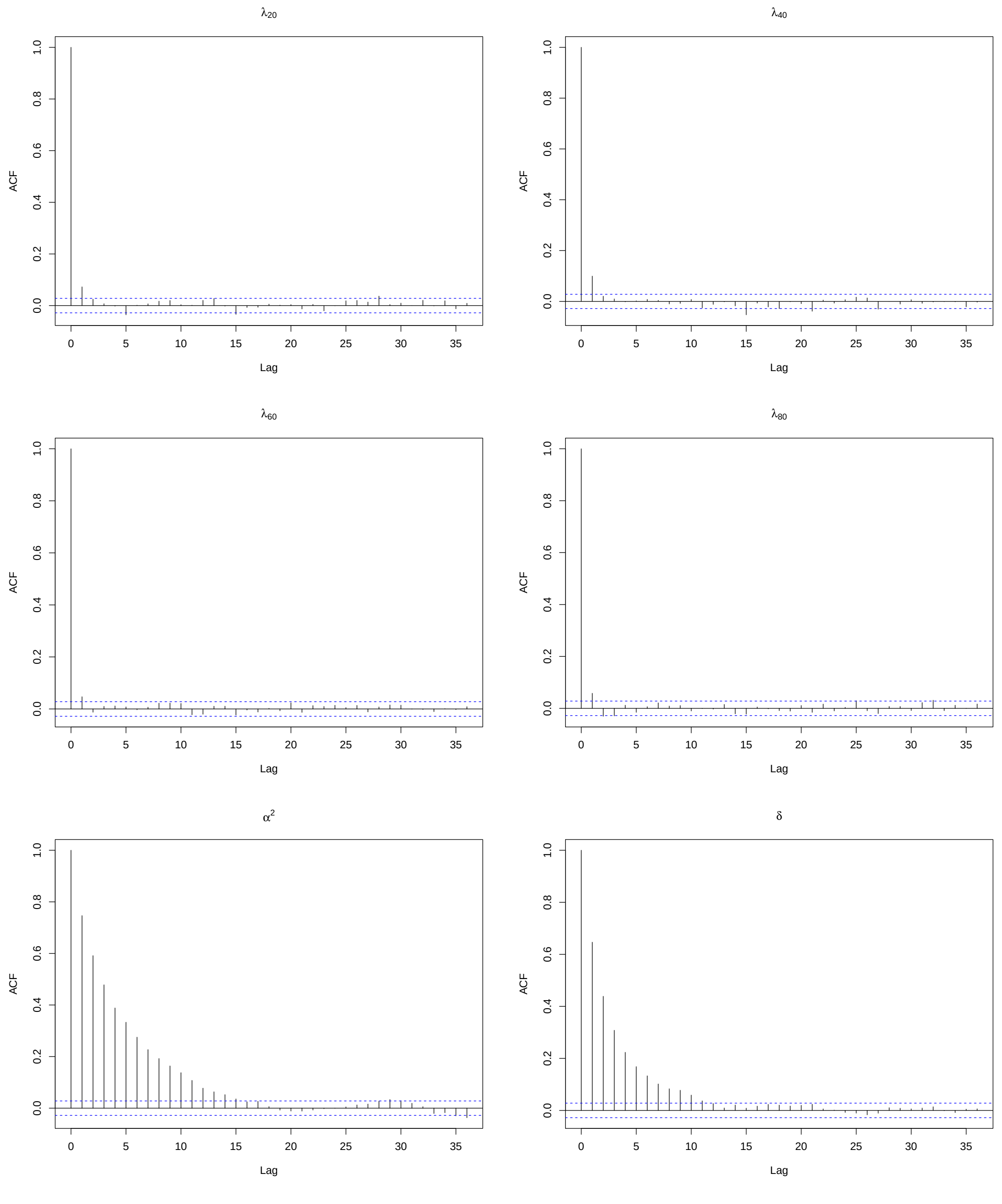

Figure 4: Autocorrelation plots for the quality parameters for wards 20, 40, 60, and 80, the prior variance parameter and the tie parameter in the South Yorkshire study. All iterations are shown.

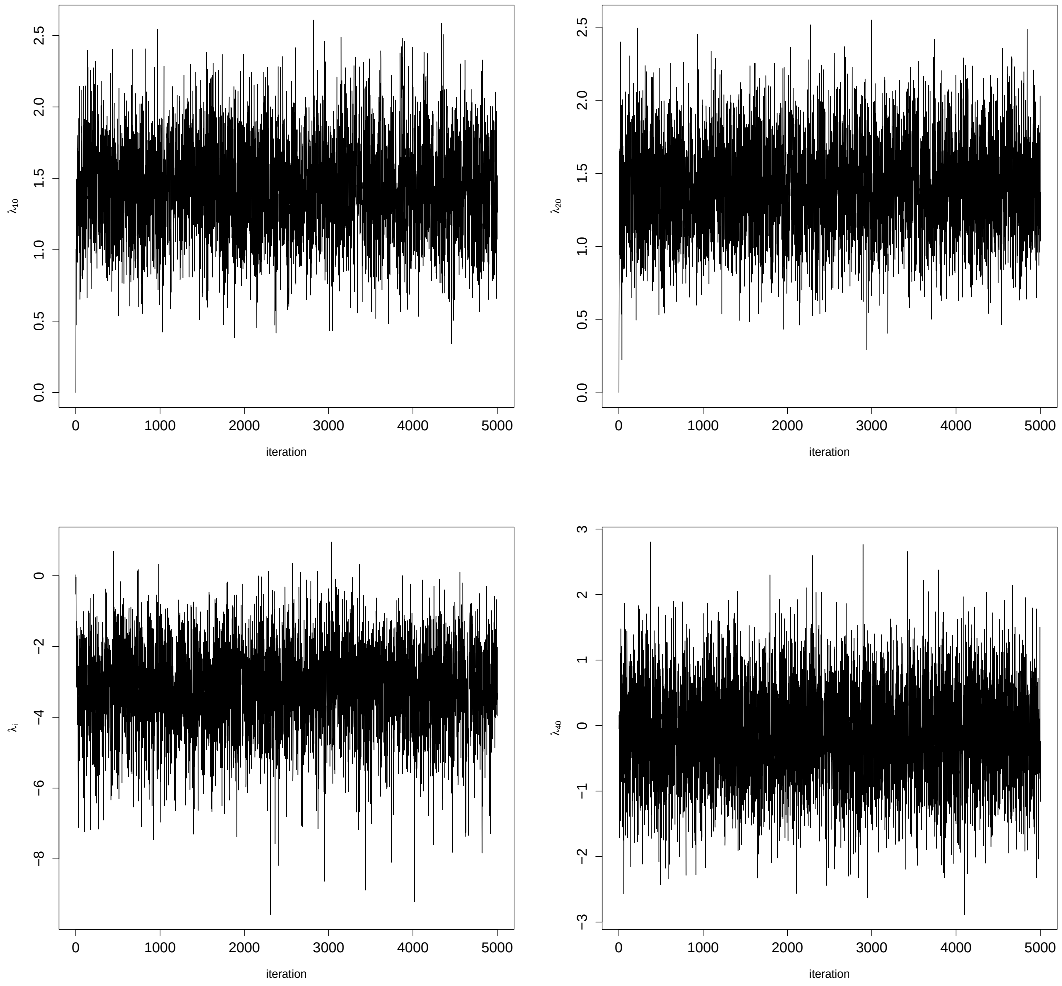

Figure 5: Trace plots for the quality parameters for wards 10, 20, 30, and 40 in the Oxfordshire study. All iterations are shown, including the burn-in period.

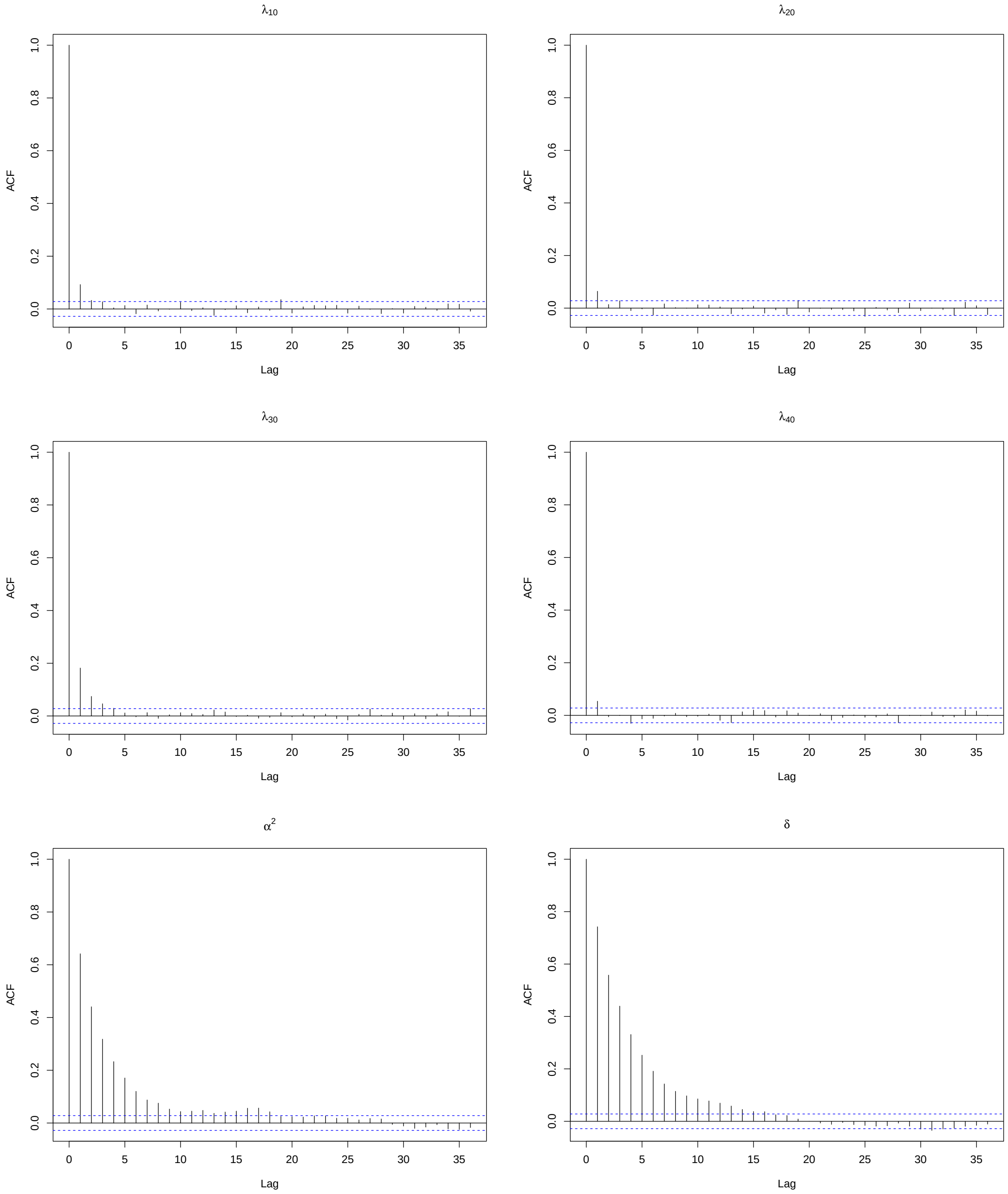

Figure 6: Autocorrelation plots for the quality parameters for wards 10, 20, 30, and 40, the prior variance parameter and the tie parameter in the Oxfordshire study.

References

[1] R.G. Seymour, D. Sirl, S.P. Preston, I.L. Dryden, M.J.A. Ellis, B. Perrat, and J. Goulding, *The Bayesian spatial Bradley–Terry model: Urban deprivation modelling in Tanzania*, Journal of the Royal Statistical Society: Series C (Applied Statistics) (2022).
