# Supplementary material for: Scalable Bayesian inference for bradley–Terry models with ties: an application to honour based abuse
Source: J Appl Stat. 2024 Dec 11;52(9):1695–712. doi: 10.1080/02664763.2024.2436608 (PMC12217112; doi:10.1080/02664763.2024.2436608)
Supplement: Mapping Risk of HBA in Oxfordshire.pdf [file CJAS_A_2436608_SM7025.pdf]

UNIVERSITY OF BIRMINGHAM

Mapping Risk of Honour Based Abuse in Oxfordshire

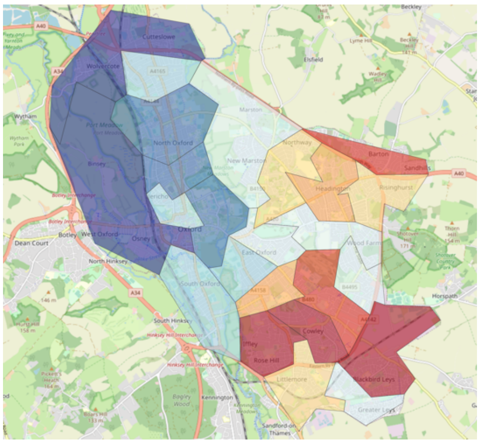

City of Oxford

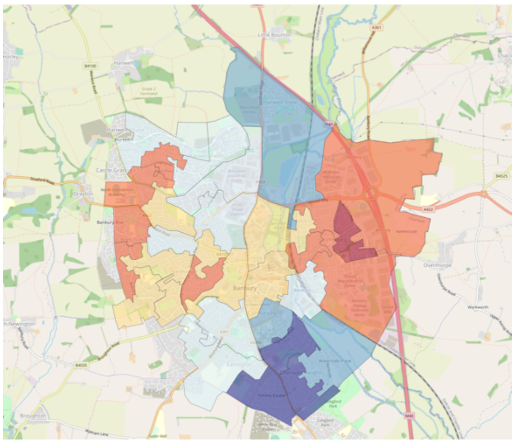

Banbury

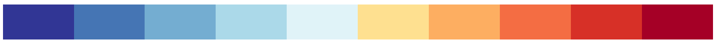

Dr Rowland Seymour

October 2023

Using a new comparative judgement method, researchers at the University of Birmingham have mapped the risk of Honour Based Abuse at community level in Banbury and the City of Oxford. The map above shows the risk of Honour Based Abuse (HBA) in each ward in Oxford and Lower Layer Super Output Areas in Banbury. Red areas have the highest risk and blue areas have the lowest risk.

To estimate the risk of HBA, the research team carried out a comparative judgement study. Comparative judgement is a new method to estimate risk of human rights abuses and has previously been used to estimate deprivation levels at local levels in developing countries and forced marriage in the UK (more information about the research methodology can be found in Seymour et. al. (2022)).

In this study, 12 experts in safeguarding against HBA from Oxfordshire were shown pairs of area and asked which of the pair has a higher prevalence of HBA. Experts were recruited with the support of Oxford Against Cutting. It is often easier to compare areas than to rank them outright or to place areas of a scale of low/high risk. The participants provided us with 766 comparisons of pairs of areas in Oxford and Banbury. From all the comparisons, it was then possible to rank the area from highest to lowest risk of HBA.

For more details contact Dr Rowland Seymour at r.g.seymour@bham.ac.uk

Overview

Honour Based Abuse (HBA) is a type of abuse used to control family members or other groups to protect supposed honour, or cultural and religious beliefs.

By collecting data from people who work in the community in Oxfordshire, researchers at the University of Birmingham have mapped data on the risks of HBA to different areas in the county.

The research team found that the South East of Oxford has a particularly high concentration of high risk wards. Grimsbury East, in Banbury, was the area with the highest risk of HBA

..

Recommendations

Based on the data and analysis, the research team recommend:

1. Agencies working in the south east of Oxford are alerted to the risk of HBA in this area.
2. Agencies working in Grimsbury are alerted to the risk of HBA in this area.
3. Further research is carried out to understand the drivers of HBA.

Risk of HBA by Local Authority

The plot below shows the risk of HBA in each area grouped by local authority. When estimating the risk of HBA, the research team set the average risk across the whole county to be zero. Areas with a large negative value have the highest risk of HBA. Areas with a large positive value have the lowest risk of HBA.

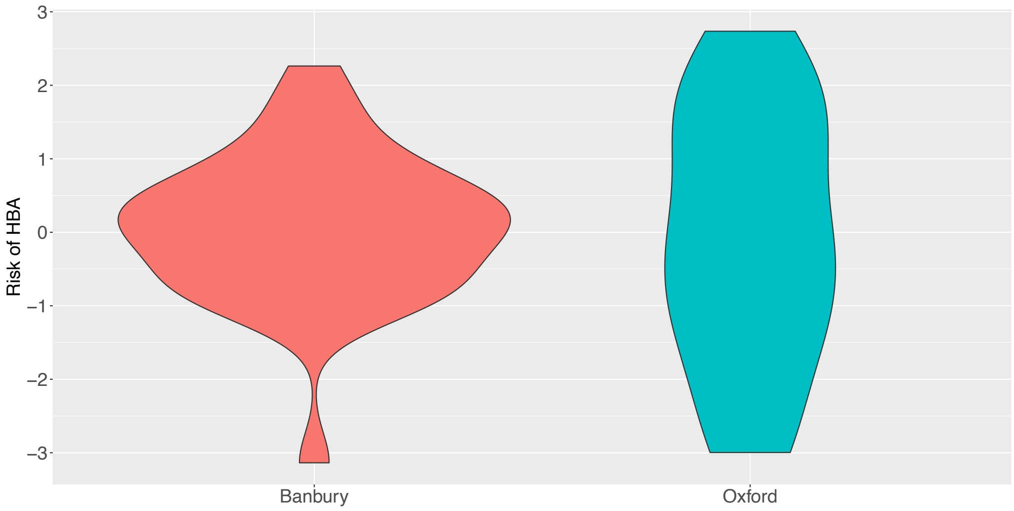

The risk of HBA in Banbury was largely the same for most areas, with the risk in each area about average compared to all the areas in the study. However, one area, Grimsbury East, had the highest risk of all areas in the study. The risk of HBA in Oxford varied from the lowest in the study to some of the highest risk areas we looked at. There is a clear spatial trend in the city, with the risk increasing the further southeast a ward is.

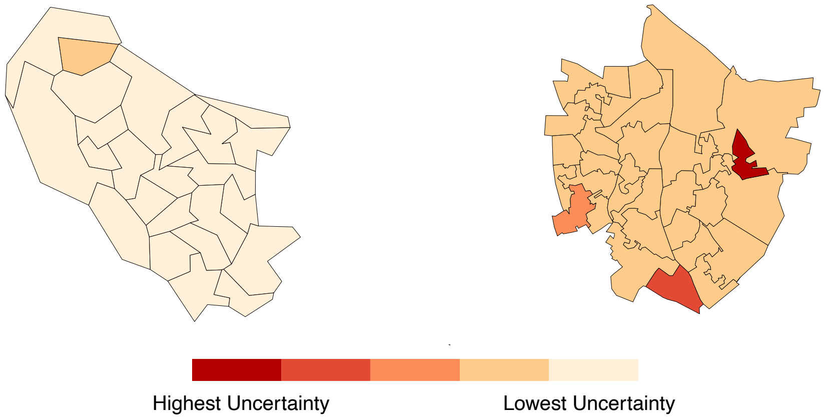

The maps above show the uncertainty in the estimates for the risk in each area. Areas shown in dark red have the highest uncertainty in their estimates, and pale-yellow wards have the lowest. For the vast majority of wards, the research team found low uncertainty in the estimates for HBA. Overall, the estimates for Banbury have slightly higher uncertainty than in Oxford because we collected more data about Oxford than Banbury. The three areas in Banbury with the higher uncertainty are areas which were chosen to have the same outcome for each comparison they were featured in. For example, each time a participant was shown a pair of areas including Grimsbury East, it was chosen as the area with the highest risk of HBA. This means we know it is a high risk area, but determining the exact level of the risk is challenging.

10 Area with the highest risk of HBA

We estimate the ten area with the highest risk of HBA are:

1. Grimsbury East
2. Cowley
3. Blackbird Leys
4. Rose Hill & Liffey
5. Temple Cowley
6. Barton & Sandhills
7. Headington
8. Donnington
9. Grimsbury South
10. Neithrop East

Why is this important?

At present, there is very little community level data about the prevalence and risk of HBA. This limits the development of local policies to tackle HBA and protect those at risk of abuse. Mapping HBA risk enables practitioners to advocate for increased funding, services, and training for professionals in the most at-risk areas to prevent and safeguard those at risk of HBA

Acknowledgements

This work was supported by the University of Birmingham School of Mathematics. Digital Research Support was provided by the Birmingham Environment for Academic Research.

Oxford Against Cutting supported with data collection.
